# Association between glucocorticoid receptor beta and steroid resistance: A systematic review

**DOI:** 10.1002/iid3.1137

**Published:** 2024-01-12

**Authors:** Md. Mojahidur Hasan, Sehreen Tory

**Affiliations:** ^1^ Rangpur Medical College University of Rajshahi Rajshahi Bangladesh; ^2^ M Abdur Rahim Medical College University of Rajshahi Rajshahi Bangladesh

**Keywords:** glucocorticoid receptor beta, steroid resistant disease

## Abstract

**Background:**

Glucocorticoids are the most commonly used anti‐inflammatory drugs for a variety of diseases, despite the fact that resistance to them is growing in a number of conditions. There is currently no biomarker that can be used to identify steroid resistance. According to a number of studies, an overexpression of the glucocorticoid receptor beta (GR‐β) isoform is associated with steroid‐resistant illness. Our goal is to find out whether or not steroid‐resistant disorders are associated with an increased level of GR‐β expression.

**Methods:**

We conducted searches in the databases of Web of Science and PubMed until January 17, 2023. This systematic review was done according to the preferred Reporting Items for Systematic Reviews and Meta‐Analyses guidelines. The Joanna Briggs Institute Appraisal scale was used to assess the quality of the included studies.

**Results:**

After the initial search, we identified 556 papers and finally included 20 studies. Twelve of these studies found an elevated level of GR‐β in the steroid resistant group. All five studies on asthma, two out of three on nasal polyps, both studies on ulcerative colitis found an up regulation of GR‐β in steroid resistant group as compared to steroid‐sensitive groups. GR‐β was also shown to be elevated in patients with allergic rhinitis, Crohn's disease and rheumatoid arthritis. In the majority of the investigations, higher levels of GR‐β were identified in peripheral blood mononuclear cells through the use of reverse transcription polymerase chain reaction.

**Conclusion:**

GR‐β was associated with steroid‐resistant diseases. It was overexpressed in steroid‐resistant diseases and has the potential to be used as a biomarker for disorders involving steroid resistance.

## Graphical abstract

Glucocorticoid receptor beta may be used as a biomarker for disorders involving steroid resistance.

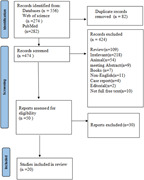

## 1 INTRODUCTION

Glucocorticoids (GCs) are the most effective anti‐inflammatory medications for the treatment of a wide variety of chronic inflammatory and immunological illnesses.^1^ Although in recent years, several novel therapies have been introduced, GC remain the first‐line treatment for long‐term control of asthma, Crohn's disease and ulcerative colitis.^2^ Despite exhibiting a favorable anti‐inflammatory impact, over 30% of people may have insensitivity to GC treatment. GC insensitivity impacts approximately 4%–10% of individuals with asthma, 30% of those with rheumatoid arthritis, almost all those with COPD, sepsis, and 10%–30% of untreated acute lymphoblastic leukemia patients.^3^ GC resistance activates the HPA axis, raising cortisol and ACTH levels. These elevated hormone levels cause a prolonged stress‐like reaction. Chronic ACTH release induces adrenal hyperplasia, an excess of GCs, mineralocorticoids, and androgens. These chemicals can cause hypertension, hypokalemia, and menstrual cycle irregularities.^4^ Individuals who are treated with GCs for a longer duration of time are more likely to acquire tissue‐specific GC resistance^5^ and It has been demonstrated that GCs, even when administered in extremely high dosages, do not have a substantial impact on chronic inflammatory and immunological conditions. These conditions include asthma, rheumatoid arthritis, inflammatory bowel disease, and autoimmune diseases.^1^ The adverse effects linked to high doses and long‐term use of GCs unfortunately limit the therapeutic advantages.^6^

GCs form a dimer when they bind to the cytosolic glucocorticoid receptor (GR), which then travels to the nucleus where it functions as a transcription factor. Through the use of alternative splicing, the primary human GR transcript can generate both the GR‐α and GR‐β, isoforms. GR‐ β acts as a dominant‐negative inhibitor of GR‐α by forming GR‐α/GR‐ β heterodimers, which then limit GR‐α's ability to induce transcriptional activation. Since GR‐ β is able to suppress the function of GR‐ α, this finding suggests that enhanced GR‐β expression might be able to modulate cell sensitivity to GCs in a wide variety of inflammatory diseases.^7^ It has been shown that a high expression of GR‐β is connected with GC insensitivity in a number of inflammatory illnesses, such as asthma, ulcerative colitis, rheumatoid arthritis, and systemic lupus erythematous.^8^

The objective of this systematic review is to comprehensively examine and analyze any possible associations between the expression of GR‐β and the development of steroid resistance in different disorders. Furthermore, it aims to explore the possible application of GR‐β as a biomarker for the detection of steroid‐resistant illnesses.

## 2 MATERIAL AND METHODS

### 2.1 Literature search

We conducted searches in the databases of Web of Science and PubMed until January 17, 2023 using the following keywords: (GR‐β) or (GRβ) or (GR β) or (GR beta) or (Glucocorticoid receptor beta) and (steroid resistant) or (steroid insensitive) or (steroid refractory) or (steroid unresponsive) or (corticosteroid resistant) or (corticosteroid insensitive) or (corticosteroid refractory) or (corticosteroid unresponsive). This systematic review was done according to the Preferred Reporting Items for Systematic Reviews and Meta‐Analyses guidelines. The PICO questions of this systematic review: Does the expression of GR‐β receptor correlate with steroid resistance in individuals who have diseases that are resistant to steroid treatment as compared to groups that are receptive to steroid treatment?

#### 2.1.1 PICO

Population: Patients suffering from conditions that were resistant to steroid treatment as well as those who were sensitive to the medication, Intervention: expression of GR‐β receptor, Comparison: Comparing GR‐β expression level between steroid resistant and steroid sensitive patients, Outcome: The association of GR‐β receptor expression in steroid resistance patients.

### 2.2 Study objectives

The objective of this study is to investigate the correlation between the expression of GR‐β in steroid‐resistant disorders and assess its potential as a biomarker.

### 2.3 Study selection and inclusion and exclusion criteria

The present study employed specific inclusion and exclusion criteria to ensure the appropriate selection of articles for analysis.

### 2.4 Inclusion criteria

1. Patients who had been diagnosed with a disease and were receiving treatment with steroids.
2. Studies divided the patients into a steroid‐resistant (SR) group and a steroid‐sensitive (SS) group.
3. A study measured the baseline GR‐β expression levels in the SR and SS groups. This could be done with immunohistochemistry, PCR, or Western blot analysis.

### 2.5 Exclusion criteria

1. Language: Non‐English articles were disregarded.
2. Excluded publication types: abstracts, animal research, reviews, systematic reviews, meta‐analyses, theses, dissertations, documents, letters, book chapters, editorials, and not full‐text.

### 2.6 Data extraction

Both the systematic search and the screening of the papers were done independently by both of the authors. Data were also extracted individually from each of the studies that were eligible, and any disagreements that arose were resolved by discussion between each of the authors. The following information was obtained from each study: the name of the author, the country where the study was conducted in, the year the study was published, the disease being studied, the sample type, the detection method, the SR sample size, and the SS sample size.

### 2.7 Quality assessment

The Joanna Briggs Institute Appraisal Checklist for Case‐Control Studies scale assessed the listed studies' quality. This tool has 10 questions about the study that should be answered “no,” “not applicable,” or “yes.” Each yes response is considered one point; therefore, the tool score is 0–10. Low‐quality studies had 0–3 points, medium‐quality ones had 4–6, and high‐quality ones had 7–10. Two reviewers classified the studies separately. Disagreements were resolved through conversation. If patients receive steroids for at least 14 days, we considered this to be an adequate amount of time for exposure.

## 3 RESULTS

### 3.1 Literature search

**Figure 1:**
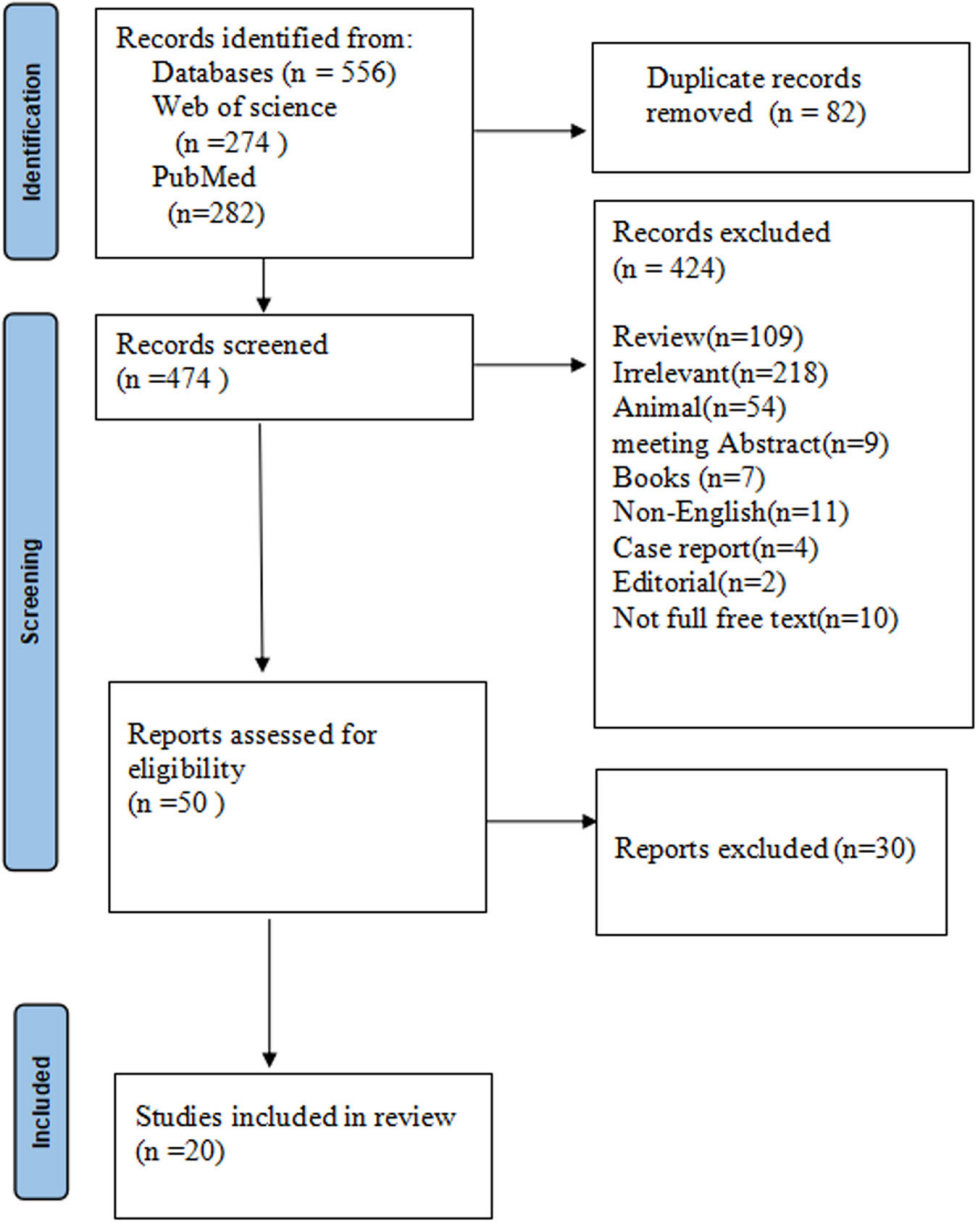
Preferred Reporting Items for Systematic Reviews and Meta‐Analyses (PRISMA) flow diagram of search strategy.

Initial searches identified a total of 556 different research. Because of the duplicate content, 82 articles were removed. After eliminating of the duplicates, we screened the remaining 474 articles by reading the titles and abstracts, and we eliminated 424 of them because 218 of them were irrelevant, 109 were reviews, 54 were about animals, 11 were written in a language other than English, 9 were meeting abstract, 10 were about not having full access to the free text, 7 were about books, 4 were about case reports, and 2 were editorials. We examined the entire texts of the remaining 50 articles, but because thirty of them did not fulfill the selection criteria, the systematic review only included a total of twenty‐articles. The search strategy used in this systematic review is shown in Figure 1.

### 3.2 Characteristics of included studies

The characteristics of the research studies that were retrieved are outlined in Table 1. The table contained information from a total of twenty different research. Five of these studies focused on asthma, three on nasal polyps, two on ulcerative colitis, two on immune thrombocytopenia, and one each on Hyper eosinophilic syndrome, allergic rhinitis, Sensorineural hearing loss, Vogt‐Koyanagi‐Harada Disease, Systemic lupus erythematosus disease, Multiple sclerosis, Rheumatoid arthritis, and Active Crohn's disease. In terms of the distribution of research based on location, six studies were carried out in China, six in the United States of America, three in Japan, and one each in Korea, Chile, the United Kingdom, Poland, and Israel. The majority of the studies (fifteen out of twenty) utilized peripheral blood mononuclear cells (PBMC) to examine the expression of GR beta.

**Table 1:** Characteristics of included studies.

Author	Country	Study period	Disease	Sample type	Method	SR sample	SS sample
[9]	USA	2012	Asthma	PBMC	RT‐PCR	11	8
[10]	USA	2010	Asthma	PBMC BAL	RT‐PCR	13 7	7 7
[11]	USA	2006	Asthma	BAL PBMC	RT‐PCR	8	7
[12]	USA	1999	Asthma	BAL, PBMC	IHC	7	8
[13]	USA	1997	Asthma	PBMC	IHC	7	6
[14]	Japan	2009	Ulcerative colitis	Colonic tissue	RT‐PCR, IHC	20	18
[15]	Japan	2002	Ulcerative colitis	PBMC	RT‐PCR	5	12
[16]	China	2021	Nasal polyps	Nasal tissue	RT‐PCR, WB	20	20
[17]	China	2010	Nasal polyps	Nasal tissue	RT‐PCR	14	26
[18]	Korea	2006	Nasal polyps	Nasal tissue	RT‐PCR	12	21
[19]	Japan	2010	Allergic rhinitis	Nasal mucosa	IHC	20	10
[20]	China	2016	Immune thrombocytopenia	PBMC	RT PCR, Western blot	10	22
[21]	China	2013	Immune thrombocytopenia	PBMC	RT PCR	10	25
[22]	USA	2019	Hyper eosinophilic syndrome	PBMC	RT qPCR	9	22
[23]⁠	China	2019	Sensorineural hearing loss	PBMC	RT PCR, WB	18	37
[24]⁠	Chile	2017	Vogt‐Koyanagi‐Harada Disease	PBMC	RT PCR	8	13
[25]⁠	China	2015	Systemic lupus erythematosus	PBMC	RT PCR, WB	14	56
[26]⁠	Poland	2008	Multiple sclerosis	PBMC	RT PCR	15	15
[27]⁠	UK	2007	Rheumatoid arthritis	PBMC	RT‐PCR	12	10
[28]	Israel	2005	Active Crohn's disease	PBMC	RT‐PCR	15	14

Abbreviations: BAL, bronchoalveolar lavage; IHC, immunohistochemistry; PBMC, peripheral blood mononuclear cells; WB, western blot.

### 3.3 Quality assessment

Table 2 presents the Critical Appraisal Checklist for case‐control studies that was developed by the Joanna Briggs Institute. This checklist is related to the quality assessment. With the exception of one study, which had a quality that was considered to be medium, all of the studies presented assessments of high quality.

**Table 2:** JBI case‐control tool for quality assessment.

Author	Q1	Q2	Q3	Q4	Q5	Q6	Q7	Q8	Q9	Q10	Quality
Author	√	√	√	√	√	√	x	√	√	√	High
[9]	√	√	√	√	√	√	x	√	x	√	High
[10]	√	√	√	√	√	√	x	√	x	√	High
[11]	√	√	√	√	√	√	x	√	x	x	High
[12]	√	√	√	√	√	x	x	√	x	√	High
[13]	√	√	√	√	√	x	x	√	x	√	High
[14]	√	√	√	√	√	x	x	√	√	√	High
[15]	√	√	√	√	√	x	x	√	√	√	High
[16]	√	√	√	√	√	x	x	√	√	x	High
[17]	√	√	√	√		x	x	√	√	√	High
[18]	√	√	√	√	√	x	x	√	x	√	High
[19]	√	√	√	√	√	x	x	√	√	√	High
[20]	√	√	√	√	√	x	x	√	x	√	High
[21]	√	√	√	√	√	x	x	√	x	x	Medium
[22]	√	√	√	√	√	x	x		x	√	High
[23]⁠	√	√	√	√	√	√	√	√	√	√	High
[24]⁠	√	√	√	√	√	x	x	√	√	√	High
[25]⁠	√	√	√	√	√	X	X	√	√	X	High
[26]⁠	√	√	√	√	√	X	X	√	x	√	High
[27]	√	√	√	√	√	√	√	√	√	√	High

*Note*: Q1. Were the groups comparable other than the presence of disease in cases or the absence of disease in controls? Q2. Were cases and controls matched appropriately? Q3. Were the same criteria used for identification of cases and controls? Q4. Was exposure measured in a standard, valid and reliable way? Q5. Was exposure measured in the same way for cases and controls? Q6. Were confounding factors identified? Q7. Were strategies to deal with confounding factors stated? Q8. Were outcomes assessed in a standard, valid and reliable way for cases and controls? Q9. Was the exposure period of interest long enough to be meaningful? Q10. Was appropriate statistical analysis used?

### 3.4 Association of GR‐β expression and steroid resistant diseases

#### 3.4.1 Asthma

All included five studies utilized PBMC to conduct their research. According to two IHC‐based investigations, GR‐β level elevated in steroid‐resistant asthma patients.^12^, ^13^ Three studies utilized RT‐PCR; two studies reported that GR‐β level was higher in PBMC of steroid‐resistant individuals,^9^, ^11^ while the other one study revealed no difference between the SR and SS groups.^10^ Two studies that examined both BAL and PBMC concluded that GR‐β level was elevated in SR asthmatics’ BAL.^11^, ^12^

No difference of GR‐α mRNA level found in PBMC of steroid resistant and sensitive group,^9^, ^10^, ^11^ also in BAL cells.^11^

#### 3.4.2 Nasal polyps

There were a total of three studies included. Both Xue et al and P. Li et al observed that GR‐β was higher in nasal tissue of corticosteroid resistant group than sensitive group.^16^, ^17^ However, Choi et al., found a different finding, which was that in the nasal tissue, GR‐β level was the same in both GC sensitive and resistant group. Choi and colleagues revealed that the GC‐resistant group had a higher level of GR‐α than the sensitive group.^18^ However, Xue and colleagues observed a decreased GR‐α in the resistant group.^16^ Additionally, Li and colleagues reported that the ratio of GR‐α/GR‐β was lower in the GC‐resistant group compared to the sensitive group.^17^

#### 3.4.3 Ulcerative colitis

Two studies^15^ and^14^ were eligible to analysis in this systematic review, and in both studies, the GR‐β mRNA level was shown to be higher in the GC‐resistant group of ulcerative colitis patients' colonic tissue and PBMC than in the GC‐sensitive group. The immunohistochemistry study also found that the GC‐resistant group had higher numbers of GR‐β + cells. In the case of GR‐α mRNA, there was not a significant difference between the GC‐sensitive and ‐resistant groups, and the immunohistochemistry experiment provided the same result.

#### 3.4.4 Immune thrombocytopenia

Liang et al. reported that the levels of GR‐α and GR‐β mRNA were lower in the PBMC of resistant group patients.^20^ Ma et al. showed that the level of GR‐α mRNA was much higher in the sensitive group, although there was no significant difference between the levels of GR‐β mRNA in the sensitive and resistant groups.^21^ Both studies could not detect GR‐β protein level.

#### 3.4.5 Crohn's disease and rheumatoid arthritis

In a study on active Crohn's disease, Towers et al. observed that the level of GR‐β mRNA in the PBMC of patients with steroid resistance was significantly higher than the level in patients with sensitive disease.^28^ Kozaci et al. found that the level of GR‐β mRNA was significantly greater in the PBMC of people with rheumatoid arthritis who were resistant to the effects of steroids, although the level of GR‐α mRNA was similar in both groups.^27^

#### 3.4.6 Other diseases

According to research carried out by Matysiak and colleagues in 2008, those with a resistant form of multiple sclerosis had lower levels of both GR‐α and GR‐β.^26^ Guan et al.,^25^ found that the levels of GR‐β and GR‐α in patients with systemic lupus erythematosus were lower in the resistant group than in the sensitive group. In patients with hyper‐eosinophilic syndrome, there was no significant difference identified between the resistant and sensitive groups' level of GR‐β mRNA expression.^22^ It was reported that there was no significant difference in the mRNA expression of GR‐α and GR‐β in patients with Vogt‐Koyanagi‐Harada disease.^24^ According to Zhang et al.'s research from 2019, patients with sensorineural hearing loss in both the SR and SS groups exhibited equal level of GR‐α and GR‐β mRNA expression.^23^ Ishida et al. found that the level of GR‐β in the nasal mucosa of steroid‐resistant individuals with allergic rhinitis was much higher than that of sensitive individuals. They did not identify any difference in GR‐α levels between the resistance group and the control group.^19^ ⁠

## 4 DESCRIPTION

Corticosteroid is the most commonly used anti‐inflammatory drug for a variety of diseases; however, 30% of patients with rheumatoid arthritis,^29^ 5%–10% of asthmatics,^30^ 29% of patients with ulcerative colitis,^31^ and 20% of patients with chron's disease^32^ either not respond to corticosteroid therapy or do not respond well to it. Alternate splicing of the GR main transcript results in the production of two isoforms of the human GR that have been designated GR‐α and GR‐β.^33^ Cotransfection studies have demonstrated that GR‐ β functions as a dominant‐negative inhibitor of GR‐ α mediated transcriptional activation through a mechanism that includes the production of GR‐ α/GR‐ β heterodimers.^34^, ^35^ It is possible that the formation of heterodimers could be responsible for the decreased efficacy of GC action in cells that overexpress GR‐β.^36^, ^37^ The mRNA for GR‐β was identified in almost every normal human tissue and cell type that was analyzed. On the other hand, it has been demonstrated that the GR‐β protein has a more limited distribution.^38^ It has been shown that a high expression of GR‐β is connected with GC insensitivity in a number of inflammatory illnesses, such as asthma, ulcerative colitis, rheumatoid arthritis, and systemic lupus erythematous.^8^

In our systematic review, Twenty studies were analyzed in the full evaluation. Twelve of these studies, which constitute 60%, found an increased level of GR‐β in the SR group compared to the SS group. In all of the five studies of asthma, higher levels of GR‐β was seen either in the PBMC or the BAL. Two out of the three studies that examined nasal polyps found that SR had a higher GR‐β, and both of the studies that noticed at ulcerative colitis found that SR had a higher GR beta.

In neither of the two studies, a higher level of GR‐β was identified in the SR group of patients with immune thrombocytopenia. GR‐β was higher in both Crohn's disease, allergic rhinitis, and rheumatoid arthritis.

According to the findings of our study, steroid‐resistant forms of asthma, nasal polyps, ulcerative colitis, allergic rhinitis, Crohn's disease and rheumatoid arthritis all had higher than normal levels of GR‐β. In most of the studies, elevated GR‐β was found in PBMC, which was detected by RT‐PCR. We observed the highest number of studies conducted on asthma and found that steroid‐resistant groups of asthma had elevated levels of GR‐β.

Within the scope of this extensive research, we compared the levels of GR‐β in steroid‐resistant illnesses. Additionally, we analyzed the difference in GR‐β levels between the SR and SS groups. Unfortunately, we were unable to find sufficient data to perform the statistical analysis. The samples that were used in the studies only consisted of a moderately low number of total participants. Though 20 studies included but small number of research was conducted for a particular disease. In case of Asthma five studies were eligible and for other disease less than three studies were included. More studies are required and there were no studies in skin disease and nephrotic syndrome where steroid is most commonly used.

In conclusion, the high expression of GR‐β in peripheral blood mononuclear cells (PBMC) shows that it has the potential to act as a biomarker for disorders that are resistant to steroids. This result is of interest due to the fact that PBMC collection is a procedure that is less intrusive compared to other approaches to tissue sampling. Clinicians and researchers may be able to acquire significant insights into the risk of steroid resistance in a variety of disorders by evaluating the GR‐β expression in patients.

More studies with larger sample sizes are needed to fully explore the diagnostic and prognostic implications of using GR‐β as a biomarker for steroid‐resistant illnesses.

## 5 CONCLUSION

There was a correlation between steroid‐resistant illnesses and the GR‐β. It has the potential to be employed as a biomarker for conditions involving steroid resistance due to the fact that it was shown to be overexpressed in diseases associated with steroid resistance.

## AUTHOR CONTRIBUTIONS

**Md. Mojahidur Hasan**: Conceptualization; Data curation; Formal analysis; Investigation; Methodology; Resources; Software; Supervision; Validation; Visualization; Writing—original draft; Writing—review & editing. **Sehreen Tory**: Conceptualization; Data curation; Formal analysis; Investigation; Methodology; Resources; Software; Writing—original draft; Writing—review & editing.

## CONFLICT OF INTEREST STATEMENT

The authors declare no conflict of interest.

## Data Availability

The authors have nothing to report.
